# *Mothers In Motion* intervention effect on psychosocial health in young, low-income women with overweight or obesity

**DOI:** 10.1186/s12889-019-6404-2

**Published:** 2019-01-14

**Authors:** Mei-Wei Chang, Susan Nitzke, Roger Brown

**Affiliations:** 1 College of Nursing, The Ohio State University, 342 Newton Hall, 1585 Neil Avenue, Columbus, OH 43210 USA; 2 Department of Nutritional Sciences, University of Wisconsin-Madison, 1415 Linden Drive, Madison, WI 53706 USA; 3 School of Nursing, University of Wisconsin-Madison, 600 Highland Avenue, Madison, WI 53792 USA

**Keywords:** Low-income women, Stress, Depressive symptoms, Obesity

## Abstract

**Background:**

*Mothers in Motion (MIM),* a community-based intervention program, was designed to help young, low-income women with overweight or obesity prevent further weight gain by promoting stress management, healthy eating, and physical activity. This paper presents the *MIM’s* intervention effect on self-efficacy to cope with stress, emotional coping response, social support for stress management, stress, depressive symptoms, and positive and negative affect.

**Methods:**

Participants (*N* = 612) were recruited from the Special Supplemental Nutrition Program for Women, Infants, and Children in Michigan. They were randomly assigned to an intervention group (410 participants) or comparison group (202 participants). During the 16-week intervention, intervention participants watched ten video lessons at home and joined ten peer support group teleconferences. Surveys with established validity and reliability were used to measure self-efficacy to cope with stress, emotional coping response, and social support for stress management. The Perceived Stress Scale, Center for Epidemiologic Studies Depression Scale, and Positive and Negative Affect Scale were used to measure stress, depressive symptoms, and positive and negative affect, respectively. A general linear mixed model was applied to test the intervention effect at the end of the 16-week intervention (T2, *n* = 338) and at three-month follow-up (T3, *n* = 311).

**Results:**

At T2, the intervention group reported significantly higher self-efficacy to cope with stress (effect size [Cohen’s *d*] = 0.53), better emotional coping response (*d* = 0.38), less stress (*d* = 0.34), fewer depressive symptoms (*d* = − 0.27), and more positive affect (*d* = 0.31) than the comparison group. However, there were no significant differences in social support for stress management and negative affect between these two groups. At T3, the intervention group still reported significantly higher self-efficacy to cope with stress (*d* = 0.32) and better emotional coping response (*d* = 0.34) than the comparison group but did not report significantly higher social support for stress management, stress, depressive symptoms, and positive and negative affect.

**Conclusions:**

To help young, low-income women with overweight or obesity manage stress, researchers and program planners may consider focusing on building self-efficacy to cope with stress.

**Trial registration:**

Clinical Trials NCT01839708; registered February 28, 2013.

## Background

Obesity is associated with numerous chronic conditions, for example, type 2 diabetes, cardiovascular disease [1], and cancer [2]. About 50% of American low-income women of childbearing age (young, low-income women) have overweight or obesity [3]. The high prevalence of overweight and obesity in this group has been associated with living in highly stressful daily life situations and poverty [4], both of which are strongly associated with more depressive symptoms [5–8]. High levels of stress and more depressive symptoms are associated with weight gain [9, 10] and negatively affect dietary intake behaviors of young, low-income women with overweight or obesity [11]; for example, food craving [9], increased intake of high-energy-dense foods [12, 13], and emotional eating [11]. Also, high levels of stress and more depressive symptoms are associated with lower levels of physical activity [11, 14, 15], less positive affect and more negative affect [16–18], and increased risk for cardiometabolic disease [19]. Therefore, it is important to develop interventions aimed to improve stress levels and depressive symptoms and to assess their effectiveness in young, low-income women with overweight or obesity.

Intervention studies that include lifestyle behaviors (healthy eating and physical activity) coupled with stress management may have great potential to help young, low-income women with overweight or obesity manage weight, thus reducing the high prevalence of overweight and obesity in this population. We collaborated with the Special Supplemental Nutrition Program for Women, Infants, and Children (WIC) in Michigan to conduct a community-based intervention study called *Mothers in Motion (MIM)*. *MIM* was designed to help young, low-income women with overweight or obesity prevent further weight gain through promotion of stress management and healthy lifestyle behaviors. *MIM’s* primary outcome was measured body weight, which has been previously published. To summarize, we did not find significant differences in body weight between the intervention and comparison groups at the end of the intervention (T2) and at three-month follow-up (T3) [20]. The secondary outcomes of *MIM* included dietary intake, which has been previously published [21]; physical activity (to be published elsewhere); and psychosocial health: stress, depressive symptoms, and positive and negative affect. The analyses for the secondary outcomes focused on examining the differences in these variables between the intervention and comparison groups. This paper presents *MIM’s* intervention effect on self-efficacy to cope with stress, emotional coping response, social support for stress management, stress, depressive symptoms, and positive and negative affect. For brevity, hereafter, self-efficacy to cope with stress is referred to as self-efficacy and social support for stress management is referred to as social support. We hypothesized that the intervention group would have greater improvements in self-efficacy, emotional coping response, social support, stress, depressive symptoms, and positive and negative affect than the comparison group.

## Methods

### Study sample

Participants (*N* = 612) were recruited from seven WIC offices in Michigan. WIC, a federally funded program, is the leading and largest public health nutrition program in the United States. In 2014, WIC nationwide served nearly 9.3 million low-income pregnant, postpartum, and breastfeeding women and children (0–5 years) [22]. WIC provides services to individuals with annual household income at or below 185% of the federal poverty line. Recruitment took place from September 2012 to January 2015. Recruiters (who were peers of the study participants) personally invited women who came to our collaborating WIC offices for their WIC appointment to be screened. Potential participants filled out a screening pencil-and-paper survey; then the peer recruiters measured their height and weight to calculate body mass index (BMI). To be eligible to participate, women were required to be not pregnant, non-Hispanic Black or white, overweight or obese (BMI 25.0–39.9 kg/m^2^), between 6 weeks and 4.5 years postpartum, 18–39 years old, free of type 1 or 2 diabetes, and able to walk more than 1 block without resting. Peer recruiters obtained written consent forms from all eligible participants. After completing a baseline interview via phone (T1), consented women returned to the WIC office where they had been recruited to be randomized to either an intervention group (410 participants) or comparison group (202 participants). The intervention participants then received ten intervention video lessons in DVD format in person and were told not to share any DVDs with anyone at any time. The comparison group received printed materials on stress management, healthy eating, and physical activity from credible websites, including some government websites (e.g., U.S. Department of Agriculture). WIC staff at our collaborating sites were not aware of participants’ randomization assignments. Detailed descriptions of recruitment and study procedures have been described elsewhere [23, 24]. Michigan State University and the Michigan Department of Community Health Institutional Review Board approved the study procedure.

### Intervention

#### Theoretical framework

Social Cognitive Theory (SCT) was used to guide the intervention design. SCT emphasizes that observing peer role models making positive behavioral changes motivates and empowers individuals to engage in positive behaviors [25]. The central concept of this theory is reciprocal determinism, which means that there is a reciprocal interaction of personal factors (e.g., self-efficacy and emotional coping response), environmental factors (e.g., social support), and behaviors (e.g., lifestyle behaviors) [25]. Our intervention addressed personal and environmental factors to promote stress management and healthy lifestyle behaviors, with the ultimate goal of preventing further weight gain. Personal factors addressed were self-efficacy (one’s confidence to perform a specific behavior) and emotional coping response. The environmental factor addressed was social support.

#### Description of intervention

A detailed description of the intervention video development has been previously published [23]. Each intervention video featured four peers of the target audience (hereafter referred to as featured women) and their family members, especially young children. The featured women modeled positive behavioral changes by demonstrating practical skills to overcoming common daily social, psychological, and environmental challenges to manage stress better, eat healthier, and be more physically active. These women and their young children were filmed at home, in a local grocery store, and in their neighborhoods over a period of one year so that we could show how they made positive changes over time. To help participants build confidence (self-efficacy), our intervention helped them identify strengths, for example, recognizing their existing skills and making small steps to change. To improve emotional coping response, we provided, for example, effective ways to identify triggers of negative emotions and to respond to those triggers. To increase social support, our intervention explained helpful strategies, for example, selecting positive social support persons and eliciting and building social support. There were four stress management video lessons. The first video lesson focused on better ways to handle daily hassles (e.g., use 5Ws [who, what, when, where, and why] and H [how]) to identify root causes of a problem, be a good mom rather than a supermom, be consistent and stay calm when feeling stressed out). Lesson two covered time saving tips for busy moms (e.g., say no and set priorities, get some tasks done at night instead of early morning, and manage time with a to-do list). Lesson three covered effective ways to handle negative feelings (e.g., breathe deeply, count to ten to stay calm, remove oneself from a troubling situation for a moment, and speak positively to oneself). Lesson four included effective ways to help with parenting (e.g., listen and talk to children, be consistent, get down to the child’s eye level to communicate with the child, and use a responsibility chart). A detailed description of lessons five to ten (healthy eating and physical activity) has been published elsewhere [21, 23].

#### Intervention implementation

A detailed description of intervention implementation has been previously published [23]. During the 16-week intervention, participants watched ten video lessons (20 min per video lesson) in DVD format at home weekly (weeks 1–4: stress management) followed by every other week (weeks 5–16: healthy eating and physical activity). After watching a designated video lesson, participants circled responses on a worksheet that asked about content of the designated video lesson watched, then mailed the worksheet to the study office using a self-addressed stamped envelope. We used the returned worksheet as an indication of watching the video lesson. Participants also joined peer support group teleconferences (30 min per session, weekly for the first four weeks, then every other week for weeks 5–16) led by moderators who were peer educators or WIC dietitians trained in motivational interviewing and group facilitation skills. We recorded attendance based on whether a participant joined a particular peer support group teleconference.

### Measures

Self-report data were collected through telephone interviews at three time points: baseline (T1), the end of the 16-week intervention (T2), and three-month follow-up (T3). We used assessments developed for the target population that have been shown to demonstrate construct validity and good internal reliability (α) to measure self-efficacy, emotional coping response, and social support [26].

#### Self-efficacy

The survey used to measure self-efficacy had ten items (α = 0.92) and asked about participants’ confidence in managing stress [26]. For example, “You can relax, even when your kids scream.” Response options were on a four-point scale ranging from 1 = not at all confident to 4 = very confident. The overall self-efficacy score was the mean of the ten-item scores, with a higher score indicating higher self-efficacy.

#### Emotional coping response

The survey used to measure emotional coping response had five items (α = 0.91) [26] and asked participants about strategies used to cope with stress. For example, “How often do you deal with or prevent stress by taking a walk?” Response options were on a four-point scale ranging from 1 = rarely or never to 4 = usually or always. The overall emotional coping score was the mean of the five-item scores, with a higher score indicating better emotional coping response.

#### Social support

The survey used to measure social support had six items (α = 0.87) [26]. For example, participants were asked “You can rely on family members, friends, coworkers, or other people for support when you need to ask for advice.” Response options were on a four-point scale ranging from 1 = rarely or never to 4 = usually or always. The overall social support score was the mean of the six-item scores, with a higher score indicating more social support.

#### Stress

The Perceived Stress Scale (nine items) with established validity and reliability was used to measure stress perception [27]. Participants were asked about their perception of stress in the past month. Response options were on a four-point scale ranging from 1 = rarely or never to 4 = usually or always. The overall stress score was the mean of the nine-item scores, with a higher score indicating lower stress.

#### Depressive symptoms

The Center for Epidemiologic Studies Depression Scale with established validity and reliability was used to measure depressive symptoms (20 items) [28]. Response options were on a four-point scale ranging from 0 = rarely or none of the time to 3 = most or all of the time. Responses to the 20 items were summed to create a depressive symptom score ranging from 0 to 60, with a higher score indicating more depressive symptoms. A score of 16 was used as a cutoff value for indication of being at risk for clinical depression [28].

#### Positive and negative affect

The Positive and Negative Affect Scale with established validity and reliability was used to measure affect (18 items) [29]. Seven items measured positive affect and 11 items measured negative affect. Response options were on a five-point scale ranging from 1 = very slightly or not at all to 4 = extremely. The overall positive affect score was the mean of the seven-item scores, with a higher score indicating more positive affect. The overall negative affect score was the mean of the 11-item scores, with a higher score indicating less negative affect.

#### Statistical analyses

Statistical analyses were conducted using NCSS Version 11 (Kaysville, UT) on 569 women (387 intervention and 182 comparison participants) after excluding 43 women who became pregnant during the study. We performed descriptive analysis on all variables, *t*-test for continuous variables, and chi-squared test for categorical variables. The outcomes of interest were self-efficacy, emotional coping response, social support, stress, depressive symptoms, and positive and negative affect at T2 and T3. To assess intervention effect, we performed a general linear mixed model for repeated measures, using baseline measures as adjusting covariates. We chose a general linear mixed model over intent-to-treat analysis because our overall retention rate for the phone interview was low for T2 (59%) and T3 (55%). A general linear mixed model is a partial intent-to-treat analysis, a method using all available data without any ad hoc imputation as in the intent-to-treat approach. Simulation studies have demonstrated that analysis with mixed models without any ad hoc imputation provides more powerful tests than mixed model analysis with last observation carried forward or imputation for intent-to-treat analysis [30]. Effect size and 95% confidence intervals were calculated using Cohen’s *d*.

## Results

### Baseline demographic characteristics

**Fig. 1:**
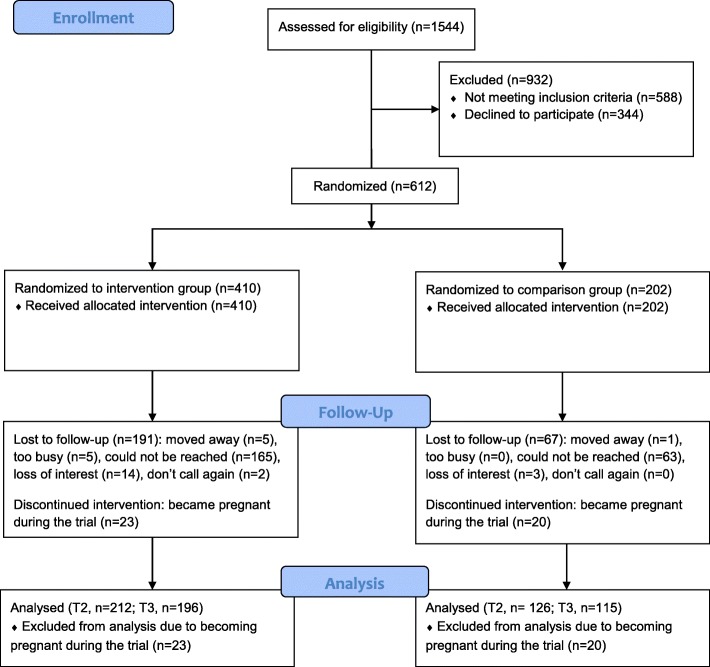
Consort chart

Figure 1 presents a Consort chart. Of women (*N* = 1544) being screened for eligibility, 956 were qualified to participate. The main reason for being excluded was a BMI outside the range of 25.0 and 39.9 kg/m^2^. Of 956 women qualified to participate, 612 were enrolled. The key reason for 344 women who did not enroll was an inability to reach them (*n* = 258) to complete the T1 phone interview. Table 1 presents the baseline demographic characteristics of the study participants. There were no significant differences in demographic variables between the intervention and comparison groups. Of the sample, 79% were white and 65% were obese. The mean age (in years) of the study sample was 28.5 ± 5.0 and mean postpartum (in years) was 1.7 ± 1.3. About 67% of the study sample had at least some college education; 23% were current smokers; 43% had a full-or part-time job; and 58% reported being at risk for clinical depression.

**Table 1:** Baseline demographic characteristics of young, low-income women with overweight or obesity (*N* = 569)

Demographic Characteristics	Intervention (*n* = 387)		Comparison (*n* = 182)	
	Mean	SD	Mean	SD
Age (years)	28.4	5.0	28.9	5.0
Postpartum status (age of the youngest child, in years)	1.6	1.2	1.9	1.3
Body mass index (BMI, kg/m^2^)	32.2	4.4	31.7	4.2
	*n*	%	*n*	%
Race				
Non-Hispanic white	306	79%	142	78%
Non-Hispanic Black	81	21%	40	22%
Current breastfeeding				
Yes	64	17%	30	16%
No	323	83%	152	84%
Education				
Some high school or less	51	13%	15	9%
High school graduate	75	19%	46	25%
Some college or technical school	186	48%	84	46%
College graduate or higher	75	19%	37	20%
Employment status				
Full-time	81	21%	44	24%
Part-time	81	21%	40	22%
Unemployed	76	20%	42	23%
Homemaker	110	28%	38	21%
Self-employed	9	2%	7	4%
Student	23	6%	8	4%
Other	7	2%	3	2%
Smoking status				
Never smoked	167	43%	93	51%
Smoked, but quit	114	30%	47	26%
Current smoker	106	27%	42	23%
Body mass index category				
Overweight (BMI 25.0–29.9)	137	35%	64	35%
Obese category I (BMI 30.0–34.9)	139	36%	71	39%
Obese category II (BMI 35.0–39.9)	111	29%	47	26%
At risk for clinical depression				
Yes (CESD score ≥ 16)	228	58.9%	105	57.5%
No (CESD score < 16)	159	41.1%	77	42.3%

*CESD* The Center for Epidemiologic Studies Depression Scale

### Intervention participation

Of intervention participants, 73% watched one of ten intervention video lessons, and 53% joined one of ten peer support group teleconference sessions [20]. In terms of watching video lessons and joining peer support group teleconference sessions for stress management, 28% did not watch any lessons; 14% watched one to three lessons; and 58% watched all four lessons. Also, 49% of participants did not join any sessions; 33% joined in one to three sessions; and 18% joined all four sessions.

### Between-group differences

Table 2 shows the intervention effect on self-efficacy, emotional coping response, and social support. The intervention group reported significantly higher self-efficacy and better emotional coping response than the comparison group at T2 and T3. However, there were no significant differences in social support between the intervention and comparison groups at T2 and T3. Table 3 presents the intervention effect on stress, depressive symptoms, and positive and negative affect. The intervention group reported significantly less stress, fewer depressive symptoms, and more positive affect than the comparison group at T2, but not at T3. There were no significant differences in reporting negative affect between these two groups at T2 and T3.

**Table 2:** Adjusted mean and standard deviation of self-efficacy, emotional coping response, and social support in young, low-income women with overweight or obesity^a^

	Intervention M (SD)	Comparison M (SD)	Effect Size	95% Confidence Interval
At baseline				
Self-efficacy	2.28 (0.71)	2.23 (0.94)	NA	NA
Emotional coping response	2.88 (0.53)	2.89 (0.50)	NA	NA
Social support	2.57 (0.76)	2.56 (0.77)	NA	NA
At the end of the 16-week intervention (T2)				
Self-efficacy**	2.56 (0.54)	2.27 (0.54)#	0.53	0.31, 0.76
Emotional coping response**	3.10 (0.39)	2.96 (0.39)	0.38	0.16, 0.61
Social support	2.65 (0.61)	2.58 (0.61)	0.10	−0.12, 0.32
At three-month follow up (T3)				
Self-efficacy**	2.54 (0.53)	2.37 (0.53)#	0.32	0.09, 0.55
Emotional coping response**	3.08 (0.38)	2.95 (0.39)	0.34	0.11, 0.57
Social support	2.57 (0.60)	2.54 (0.60)	0.06	−0.17, 0.29

^a^Self-efficacy = self-efficacy to cope with stress. Social support = social support for stress management. Score ranged from 1 to 4 for each variabl; the higher socore, the better. Baseline: *N* = 569 (387 intervention, 182 comparison). T2: *n* = 338 (212 intervention, 126 comparison). T3: *n* = 311 (196 intervention, 115 comparison). **p ≤ 0.01 for comparisons between the intervention and comparison groups within a timeframe. #*p* ≤ 0.05 for comparisons between T2 and T3 within the comparison group

**Table 3:** Adjusted mean and standard deviation for stress, depressive symptoms, and positive and negative affect between the intervention and comparison participants who were young, low-income women with overweight or obesity

	InterventionM (SD)	ComparisonM (SD)	Effect Size	95% Confidence Interval
At baseline				
Stress	2.55 (0.62)	2.58 (0.38)	NA	NA
Depressive symptoms	19.85 (10.47)	19.86 (10.00)	NA	NA
Positive affect	3.32 (0.76)	3.22 (0.72)	NA	NA
Negative affect	3.71 (0.72)	3.69 (0.77)	NA	NA
At the end of the 16-week intervention (T2)				
Stress**	2.56 (0.358)	2.44 (0.36)#	0.34	0.11, 0.56
Depressive symptoms*	17.45 (8.38)	19.68 (8.35)#	−0.27	−0.49, − 0.04
Positive affect*	3.47 (0.64)	3.27 (0.64)	0.31	0.09, 0.53
Negative affect	3.92 (0.57)	3.80 (0.45)	0.23	0.00, 0.45
At three-month follow-up (T3)				
Stress	2.55 (0.36)	2.51 (0.36)#	0.11	−0.12, 0.34
Depressive symptoms	17.56 (8.32)	18.04 (8.30)#	−0.06	−0.29, 0.17
Positive affect	3.43 (0.63)	3.30 (0.63)	0.21	−0.03, 0.44
Negative affect	3.96 (0.71)	3.88 (0.57)	0.12	−0.11, 0.35

Baseline: *N* = 569 (387 intervention, 182 comparison). T2: *n* = 338 (212 intervention, 126 comparison). T3: *n* = 311 (196 intervention, 115 comparison). Stress (score ranged 1–4): the higher the score, the less stress. Depressive symptoms (score ranged 0–60): the lower the score, the fewer depressive symptoms. Positive affect (score ranged 1–4): the higher the score, the more positive affect. Negative affect (score ranged 1–4): the higher the score, the less negative affect. *p < 0.05, **p ≤ 0.01 for comparisons between the intervention and comparison groups within a timeframe. #*p* < 0.05 for comparisons between T2 and T3 within the comparison group

### Within-group differences

Between T2 and T3, the intervention group had no significant change in self-efficacy, but the comparison group reported significant improvement in self-efficacy (mean difference = − 0.09, *p* = 0.04). There were no significant changes in emotional coping response and social support between T2 and T3 for both groups (Table 2). In terms of stress, depressive symptoms, and positive and negative affect (Table 3), the intervention group had no significant changes in stress and depressive symptoms, but the comparison group reported significantly less stress (mean difference = − 0.07, *p* = 0.04) and fewer depressive symptoms (mean difference = 1.65, *p* = 0.05) between T2 and T3. There were no significant changes in positive and negative affect between T2 and T3 for both groups.

### Completers versus non-completers

#### Baseline demographic characteristics

The overall retention rate for completing the phone interview at T3 was 55%: 51% in the intervention group and 63% in the comparison group. There were significant differences in baseline demographic characteristics between completers and non-completers. Compared to non-completers, completers were older, had lower BMI, were currently breastfeeding, had a college education or higher, were homemakers, and were less likely to be a current smoker. There were no significant differences in postpartum status and race.

#### Baseline self-efficacy, emotional coping response, social support, stress, depressive symptoms, and positive and negative affect

Compared to non-completers, completers reported significantly higher social support but fewer depressive symptoms at baseline. There were no significant differences in baseline self-efficacy, emotional coping response, stress, and positive and negative affect between the completers and non-completers.

### Stress management video dose response in relation to intervention effect

For the main analysis, we included women who completed the phone interview regardless of how many video lessons they watched (dose response). Later, we explored the stress management video dose response in relation to intervention effect by comparing those who watched all four video lessons and who completed the study vs. those who were assigned to the comparison group and who completed the study. The results showed no changes, as described above (between-group differences and within-group differences).

## Discussion

*MIM* was a large community-based lifestyle behavior weight management intervention program coupled with stress management for young, low-income women with overweight or obesity. We found that *MIM* effectively helped young, low-income women with overweight or obesity improve self-efficacy and emotional coping response but not social support in the short term. Also, the intervention effectively helped the intervention participants reduce stress and depressive symptoms and improve positive affect in the short but not long term.

This was the first weight management intervention study for young, low-income women with overweight or obesity that reported intervention effect on self-efficacy, emotional coping response, and social support for stress management. Therefore, we are unable to compare our study findings to others. Although the *MIM* intervention provided tips for and encouraged women to elicit social support, there was no detectable effect on social support. During the intervention phase, we listened to at least 25% of randomly selected audio recordings of the peer support group teleconferences for intervention fidelity monitoring. We learned that some women already had strong social support systems and others had minimum or no social support. We also learned that many women did not feel comfortable asking for help for various reasons. For example, some worried about being judged or rejected. Others worried that information shared would not be kept confidential. Also, some women’s support people were going through challenging times. We observed that intervention participants openly shared their personal challenges with their peers because our peer support group teleconference was private and confidential. Unfortunately, our peer support group teleconferences were scheduled based on the moderators’ availability, a poor match to the participants’ schedules; thus, many women were not able to join in.

Previous lifestyle intervention studies that included stress management for young, low-income women with overweight or obesity did not find significant differences in stress [31, 32] or depressive symptoms [32] between the intervention and control groups. However, we found that the *MIM* intervention group reported significantly less stress, fewer depressive symptoms, and more positive affect than the comparison group in the short term. The differences in findings between the previous [31, 32] and present studies might be due to variations in intervention content, modes of delivery, and sequence of intervention messages. While previous studies requested that intervention participants attended in-person group meetings [31, 32], our intervention participants watched *MIM* videos in DVD format at home and joined peer support group teleconferences. Also, previous studies emphasized stress and time management [31] or progressive relaxation and deep breathing [32]. Our intervention included in-depth content about handling common daily challenges for the target audience to make positive changes. Moreover, while the previous studies delivered lifestyle intervention and stress management content simultaneously [31, 32], we applied a sequential approach based on feedback from the target audience and our previous observations with this population. Young, low-income women with overweight or obesity consistently expressed the need to manage their stress before they would seriously consider eating healthier or being more physically active.

Our results revealed that *MIM*’s intervention effect on self-efficacy, emotional coping response, stress, and depressive symptoms were maintained between T2 and T3. However, our comparison group also reported significant improvements in self-efficacy, stress, and depressive symptoms between T2 and T3. We strongly believe that the findings of the intervention group were not affected by watching the physical activity video lesson, because only a small number of them watched it. Also, our preliminary analysis did not reveal any differences in physical activity between groups and within group. Nevertheless, our results suggest the importance of increasing self-efficacy to help young, low-income women with overweight or obesity reduce stress and depressive symptoms. The unexpected findings (positive improvements in the comparison group between T2 and T3) are perhaps due to intervention contamination. For example, intervention participants shared videos in DVD format with the comparison participants. At the T2 phone interview, intervention participants were asked if they had shared the *MIM* videos with women who were assigned to the comparison group (Yes/No); approximately 8% reported ‘yes’ [20]. Unfortunately, we did not collect contamination data at T3; thus, the extent of intervention contamination remains unknown. Also, comparison participants received all ten intervention video lessons when they completed the final data collection per our agreement with State of Michigan WIC. This approach might have impacted the study findings. The contamination occurrence in the present study is perhaps due to a perceived value of the video based on numerous reported anecdotes. Another potential explanation is that our videos included stress management. To our knowledge, there are limited resources available to help young, low-income women manage their daily stress and hassles.

The *MIM* program was delivered via video and peer support group teleconference. We would recommend the use of video that features peers of target audience demonstrating practical skills to overcome daily challenges to make positive changes. Our recommendation is supported by the fact that the all *MIM* video lessons have been implemented by WIC nationwide to supplement their daily practice. However, we would not recommend the use of peerp support group teleconference for the target population because of high cost in training personal to lead the sessions and logistic issues, for example, difficulty grouping women and scheduling the teleconference, frequent time conflicts with women’s busy schedules, and disconnected phones.

The study has limitations. Our sample may not be representative of the broader population of young, low-income women with overweight or obesity. We enrolled a high proportion of women who were at risk for clinical depression (58%), which may be an indication of a lack of resources available in the community to help the target audience manage stress and alleviate depressive symptoms. Also, most of our Black participants were recruited from severely economically depressed areas with high crime rates. Moreover, the intervention contamination at T3 appears to have potentially affected the study findings. Yet intervention participants sharing videos with comparison group participants suggests that this peer led video is of interest to young, low-income women with overweight or obesity. Finally, *MIM* was not powered to detect the difference in stress and depressive symptoms, but rather the primary outcome of body weight between the intervention and comparison groups. Future studies are needed to confirm the study findings.

## Conclusions

In summary, the *MIM* stress management intervention effectively helped young, low-income women with overweight or obesity improve self-efficacy and emotional coping response in both the short and long term. Also, the intervention reduced the target audience’s stress and alleviated depressive symptoms in the short term but not in the long term. Future studies may consider including peers of the target audience to demonstrate effective strategies to manage daily stress. Finally, researchers and program planners conducting stress management intervention studies for the target population may consider including strategies to boost self-efficacy to cope with stress.

## Abbreviations

BMI: Body Mass Index
MIM: Mothers in Motion
WIC: The Special Supplemental Nutrition Program for Women, Infants, and Children
